# Non-contrast T_1_ and T_2_ relaxometry characterizes reperfusion injury of acute MI in swine

**DOI:** 10.1186/1532-429X-17-S1-W14

**Published:** 2015-02-03

**Authors:** Haiyan Ding, Michael Schär, Karl H Schuleri, Henry R Halperin, M Muz Zviman, Roy Beinart, Daniel A Herzka

**Affiliations:** 1 Center for Biomedical Imaging Research, Department of Biomedical Engineering, Tsinghua University, Beijing, China; 2 Department of Biomedical Engineering, Johns Hopkins School of Medicine, Baltimore, MD, USA; 3 Russell H. Morgan Department of Radiology and Radiological Science, Johns Hopkins School of Medicine, Baltimore, MD, USA; 4 Department of Medicine, Cardiology, Johns Hopkins School of Medicine, Baltimore, MD, USA; 5 Department of Radiology, Mercy Fitzgerald Hospital, Darby, PA, USA; 6 Heart Institute, Sheba Medical Center, Tel Aviv University, Ramat Gan, Israel

## Background

Reperfusion injury in acute myocardial infarction (MI) results in edema, necrosis, microvascular obstruction (MVO), and intramyocardial hemorrhage (IMH), the latter presents an interesting clinical target. [1] Cardiovascular MRI has been shown capable of characterizing all of these tissue components. Other than MVO, which is currently detected by flow-deficient regions in contrast enhanced imaging, all other tissue components can be identified by T_1_ and T_2_ (T_2_*). Theoretically, the byproducts of blood breakdown observed with IMH lead to decreased T_1_ and T_2_ (T_2_*). [2] Conversely, free water accumulation (edema) and necrosis lead to increased T_1_ and T_2_. [2] Hence, direct and quantitative measurement of relaxation rates is promising in myocardial tissue characterization, avoiding ambiguity typical of weighted images (i.e. T_2_-weighted spin-echo), undesired signal loss from T_2_* (weighted) images or the uncertainty introduced by contrast agent kinetics. *Hypothesis*: Combined T_1_ and T_2_ mapping can characterize reperfused MI without contrast agents.

## Methods

MI was induced in swine by 1 (N=3) or 2 (N=3) hr balloon occlusion of the LAD after the first diagonal, with MRI 7-9 days post MI (Achieva TX, Philips). Relaxometry: 3D respiratory navigator-gated T_2_-mapping [3]; 2D Breath-hold T_1_-mapping (MOLLI) [4]. Clinical standard: breath-hold black-blood T_2_W TSE (BB-T_2_-STIR) [5]; early (3 min post) gadolinium-enhanced images (EGE) using PSIR and 0.2 mmol/kg Magnevist. [6]. IMH was identified in T_2_W images/T_1_/T_2_ maps as areas of hypointensity surrounded by hyperintense signal/T_1_/T_2_ representing edema. MVO was defined in EGE images as hypointense areas surrounded by enhanced MI. The co-localization of tissue types among techniques was examined.

## Results

**Figure 1:**
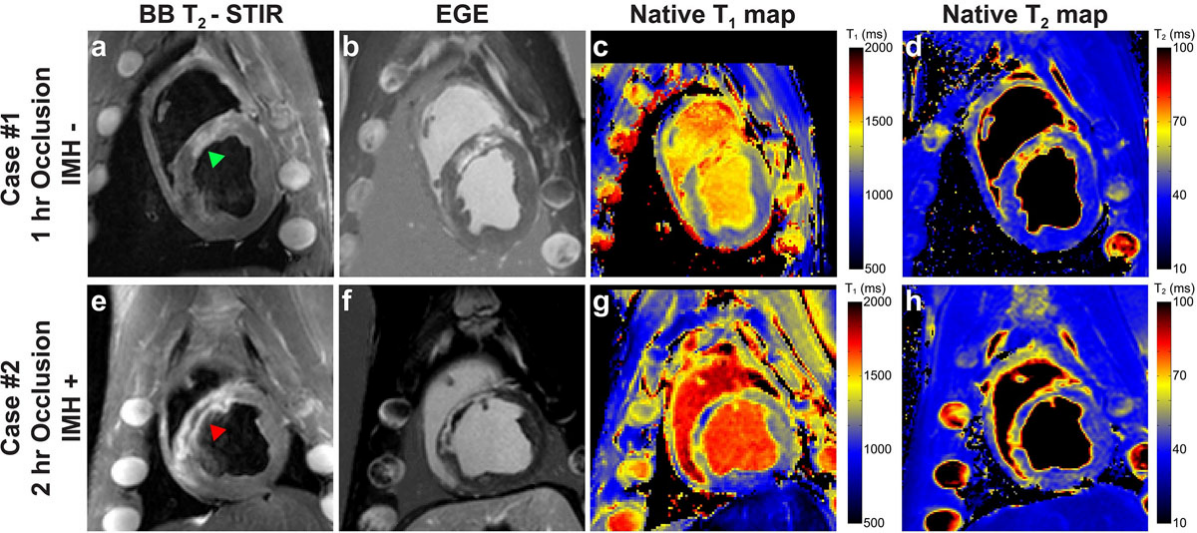
Matched representative SAX images from swine without hemorrhage after 1 hr LAD occlusion (Case #1) (a-d) and with hemorrhage after a 2 hr occlusion (Case #2) (e-h). In Case #1, significant T_1_ and T_2_ elevation are present in both maps (c,d), though no MVO was observed with EGE (b) nor was a hypointense core present in T_2_W images (a). In comparison, Case #2 shows clear IMH, demarked by decrease in T_1_ and T_2_, surrounded by edema, shown by increased T_1_ and T_2_ (g and h), all of which are excellently co-localized with that in T_2_W (e) and EGE (f). Green arrowhead indicates edema; red arrowhead indicates the core of IMH. Note that T_1_ mapping is influenced by off-resonance and lower image resolution. As the result of competing effects on T_1_ and T_2_ from IMH and edema, partial volume averaging makes the T_2_ in IMH close to that of normal myocardium.

IMH was detected in all animals with 2 hr occlusions, identified by decreased T_1_ and T_2_, and was spatially consistent with the hypoenhanced core in BB-T_2_-STIR and with MVO in EGE. Edema was observed in all animals (elevated T_1_and T_2_). (Fig. 1)

**Figure 2:**
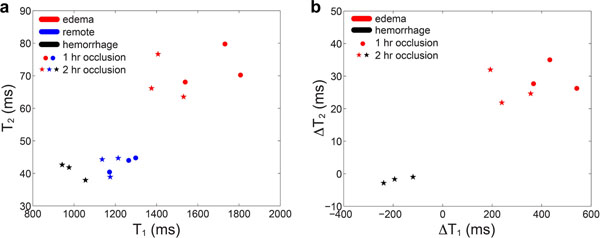
a. ROI-based T_1_ & T_2_ for edema, IMH and remote myocardium from matched T_1_ and T_2_ maps. b. Changes in T_1_ & T_2_ after subtraction of the reference values of remote myocardium. Edema had higher T_2_ than both remote myocardium or IMH. T_1_ can be used to discriminate between edema, IMH and normal myocardium, though the distributions may overlap. Edema and IMH can be classified with higher specificity using both T_1_ and T_2_.

Planimetry showed that relative to remote myocardium, T_1_ and T_2_ of edema were significantly higher (p < 0.001 and p <1e-5, respectively), while within IMH T_1_ was lower (p = 0.001) and T_2_ the same (p = 0.28). (Fig. 2)

## Conclusions

Though either T_1_ or T_2_ can be used to separate tissues, combined T_1_ and T_2_ mapping may allow for more accurate detection of IMH in reperfusion injury, without variability from contrast kinetics, or BB-T_2_-STIR artifacts. [7] Based on a small number of animals, T_2_ was superior in edema detection, while T_1_ performed better in IMH detection. Combined relaxometry may identify tissues with better specificity than individual and may help clarify the link between MVO and IMH. High-resolution relaxometry may be necessary to avoid partial volume.

## Funding

Funded in part by the American Heart Association - 11SDG5280025.
